# Theory on the Coupled Stochastic Dynamics of Transcription and Splice-Site Recognition

**DOI:** 10.1371/journal.pcbi.1002747

**Published:** 2012-11-01

**Authors:** Rajamanickam Murugan, Gabriel Kreiman

**Affiliations:** 1 Department of Biotechnology, Indian Institute of Technology Madras, Chennai, India; 2 Children's Hospital Boston, Harvard Medical School, Boston, Massachusetts, United States of America; 3 Swartz Center for Theoretical Neuroscience, Harvard University, Cambridge, Massachusetts, United States of America; 4 Program in Biophysics, Program in Neuroscience, Harvard Medical School, Boston, Massachusetts, United States of America; Center for Genomic Regulation, Spain

## Abstract

Eukaryotic genes are typically split into exons that need to be spliced together to form the mature mRNA. The splicing process depends on the dynamics and interactions among transcription by the RNA polymerase II complex (RNAPII) and the spliceosomal complex consisting of multiple small nuclear ribonucleo proteins (snRNPs). Here we propose a biophysically plausible initial theory of splicing that aims to explain the effects of the stochastic dynamics of snRNPs on the splicing patterns of eukaryotic genes. We consider two different ways to model the dynamics of snRNPs: pure three-dimensional diffusion and a combination of three- and one-dimensional diffusion along the emerging pre-mRNA. Our theoretical analysis shows that there exists an optimum position of the splice sites on the growing pre-mRNA at which the time required for snRNPs to find the 5′ donor site is minimized. The minimization of the overall search time is achieved mainly via the increase in non-specific interactions between the snRNPs and the growing pre-mRNA. The theory further predicts that there exists an optimum transcript length that maximizes the probabilities for exons to interact with the snRNPs. We evaluate these theoretical predictions by considering human and mouse exon microarray data as well as RNAseq data from multiple different tissues. We observe that there is a broad optimum position of splice sites on the growing pre-mRNA and an optimum transcript length, which are roughly consistent with the theoretical predictions. The theoretical and experimental analyses suggest that there is a strong interaction between the dynamics of RNAPII and the stochastic nature of snRNP search for 5′ donor splicing sites.

## Author Summary

The DNA encoding most eukaryotic genes is interrupted by long sequences called introns. These introns need to be removed through the process of splicing to produce the mature messenger RNA. The process of splicing plays a critical role in determining the exact aminoacid content of the ensuing protein. Several molecules denominated small nuclear ribonucleo proteins (snRNPs) are involved in finding the appropriate 5′ donor splicing sites for splicing. Transcription and splicing occur simultaneously and the ultimate product depends on the relative speed of transcription and the stochastic dynamics underlying splicing. Here we propose a biophysically plausible theory that describes the ongoing interactions between transcription and splicing. We show that the theoretical predictions are consistent with experimental measurements of the abundance patterns of different exons and transcripts across tissues.

## Introduction

Transcription of eukaryotic genes by the RNA polymerase II complex (RNAPII) produces a primary mRNA transcript (pre-mRNA) that contains both exons and introns. Introns are removed by splicing [1], [2], [3] via the assembly of a spliceosomal complex including small nuclear ribonucleo proteins (snRNPs) [4], [5], [6], [7]. Recent studies show that the majority of genes in higher eukaryotes are alternatively spliced and, therefore, contribute significantly to the structural as well as functional complexity and diversity of organisms [8], [9], [10]. The process of splicing can start as soon as the pre-mRNA begins to emerge from RNAPII. *Cis*-acting regulatory elements such as splicing enhancers and silencers generally determine the splicing pattern of a given multi-exonic gene especially when transcription is not kinetically coupled to the splicing [11], [12], [13], [14]. However, when transcription is coupled to splicing, inclusion or exclusion of an exon in the final transcript will also be strongly influenced by the transcription elongation rate as well as the local concentrations of various factors involved in the spliceosomal assembly and their interactions [15], [16], [17], [18].

Two basic models have been proposed to explain the various differences in the alternative splicing patterns of a given gene. According to the kinetic model [19], inclusion or exclusion of an exon in the final transcript is determined by the transcriptional elongation rate associated with the corresponding pre-mRNA in addition to the *cis*-acting regulatory elements. Exons are classified as ‘strong’ or ‘weak’ depending on whether they possess *cis*-acting regulatory elements associated with them or not. The inclusion of ‘strong’ exons is favored at higher transcriptional elongation rates whereas ‘weak’ exons may be included in the final transcript only when the transcriptional elongation rate is comparatively slower. Since the concentration of snRNPs in the vicinity of the transcriptional machinery is fixed under steady state conditions, a strong exon that has emerged recently from the transcriptional assembly will have a better chance of interacting with the snRNPs as compared to a weak exon that emerged earlier. Therefore, a weak exon will have a better chance to interact with the snRNPs only when there is a decrease in the rate or a pause in the transcriptional elongation process. According to the recruitment model [20], inclusion or exclusion of an exon is also decided by the interaction of the C-terminal domain (CTD) of RNAPII with a set of gene and exon specific DNA binding proteins and the snRNPs [19], [20] in addition to *cis*-acting regulatory elements. The CTD of the RNAPII interacts directly with the snRNPs and other factors, increasing the local concentrations of these factors in the vicinity of the emergence of a weak exon and thus enhancing the probability of weak exons to interact with the snRNPs.

There are four basic variables involved in the definition of an exon: (1) *cis*-acting regulatory elements [11], [12], [13] (2) transcription elongation rate [19] (3) interactions between the CTD of RNAPII and the snRNPs, hnRNPs and SR proteins [19], [20] (often referred to as ‘recruitment’) and (4) the stochastic dynamics involved in the recognition of the 5′ donor splice sites by U1 snRNPs while the pre-mRNA is evolving from the transcription assembly. Variables 1 and 3 are specific to each exon whereas variables 2 and 4 are generic and affect all the exons across various transcripts of an organism.

Most of the current splice pattern prediction algorithms consider mainly the *cis*-acting regulatory elements (variable 1) [21], [22], [23], the kinetic model focuses on variable 2 [19] and the recruitment model considers mainly variable 3 [19], [20]. None of the current algorithms or models considers the stochastic dynamics associated with the snRNP search process (variable 4). Here we propose a biophysically plausible theory from first principles to describe the coupled dynamics of transcription and splicing. This work presents initial steps towards capturing the basic relationship between transcriptional elongation and splicing; the simplified model that we propose does not include multiple critical components that affect the splicing outcome including *cis*-acting pre-mRNA sequence motifs, *trans*-acting interactions with different proteins and variable rates of RNAPolII transcription. We focus on the stochastic dynamics whereby snRNPs locate the 5′ donor sites and how this search influences the outcome of splicing. We evaluate the theoretical predictions by analyzing expression data at the exon level from exon microarrays and RNAseq experiments across different tissues in mice and humans.

## Results

### A theoretical framework of coupled transcription and splicing

**Figure 1:**
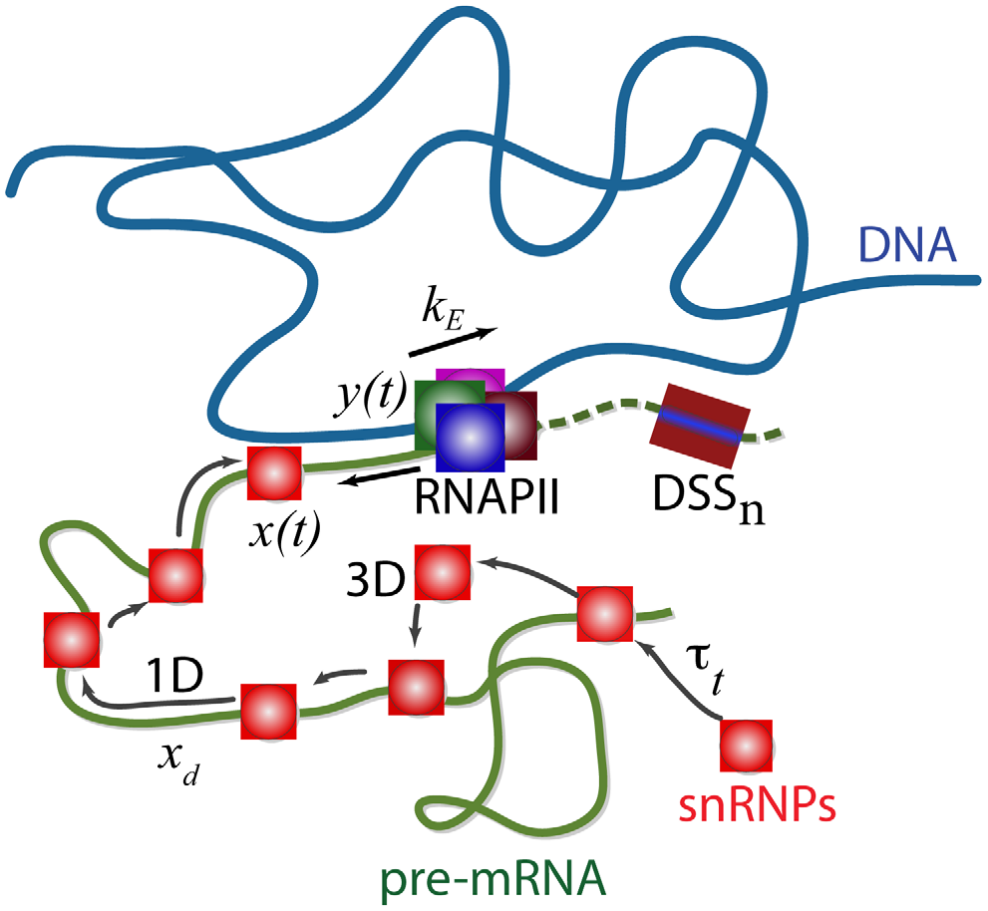
Schematic description of the various simultaneous processes that take place when splicing is coupled to transcription. In this scheme, the RNAPII complex has already initiated transcription and is currently in the transcriptional elongation step with an elongation rate 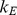 (bases s^−1^). The RNAPII complex is located at position *y(t)* on the pre-mRNA chain. The snRNPs can locate the 5′ donor splicing site (DSS_n_) at position *n* either via a pure three-dimensional diffusion process or via a combination of three- and one-dimensional diffusion. Here the snRNP has already non-specifically bound the pre-mRNA and is shown scanning the pre-mRNA at position *x(t)*. DSS_n_ has not been transcribed yet in this scheme.

Recent single cell studies have revealed [24], [25], [26] that small nuclear ribonucleoproteins (snRNPs) and other splicing proteins are freely diffusing inside the entire volume of various nuclear and splicing factor compartments of within the eukaryotic cell nucleus. Splicing is kinetically coupled to transcription when the time required to generate a complete transcript is longer than the time required for the assembly and catalytic activity of the spliceosomal proteins. Under such coupled conditions, we must simultaneously consider at least two different types of dynamical processes: (i) transcription elongation by the RNA polymerase II transcription complex (RNAPII) and (ii) the search process whereby snRNPs locate the 5′ donor splicing sites (DSS) on the emerging pre-mRNA to initiate the spliceosomal assembly (**Figure 1**). The freely diffusing U1 snRNP can locate the donor splicing sites via two different types of mechanisms: a pure three-dimensional diffusion-controlled collision route (3D) and a combination of three-dimensional and one-dimensional diffusion dynamics as in the case of typical site-specific DNA-protein interactions (3D+1D) [27], [28], [29], [30]. Upon successful binding of the U1snRNP molecule to the 5′ donor site, a cascade of molecular processes involving multiple snRNPs ensues, culminating in the formation of the spliceosomal complex and intron removal [1], [2], [3]. Except for the binding of U1 snRNPs at the 5′ donor site, all the other steps involve the hydrolysis of ATPs. This means that the binding of U1 is a purely thermally driven process and here we focus on the dynamics involved in this rate-limiting step. All the other binding events and reactions, including transcription elongation, involve ATP hydrolysis and we therefore assume that the effects of thermal induced fluctuations are minimal in these reaction steps. We ignore the thermal induced fluctuations over these reaction steps while describing the search dynamics of snRNPs along the pre-mRNA. The overall probabilities associated with the interaction of snRNPs with various DSSs depend on the type of search mechanism followed by the snRNPs.

We start by considering the model illustrated in **Figure 1** where the U1 snRNP has bound the emerging pre-mRNA via non-specific interactions facilitated by 3D diffusion and it scans the concomitantly emerging pre-mRNA for the presence of DSSs via 1D diffusion. At a given time *t*, let *y(t)* denote the length of the emerging pre-mRNA and let *x(t)* denote the position of the non-specific bound U1 snRNP on the pre-mRNA chain. The DSS under consideration is located at position *x = n* (DSS_n_), which has not been transcribed at time *t* (or is currently not reachable by the snRNP due to steric hindrance). Such coupled dynamics of snRNPs and RNAPII, represented by the set of dynamic position variables *x* and *y* (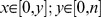) on the same pre-mRNA, can be described by the following set of Langevin type stochastic differential equations [31]:
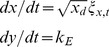
(1)The transcription elongation rate is denoted as *k_E_* (bases s^−1^). *x_d_* (bases^2^s^−1^) is the 1D diffusion coefficient associated with the searching dynamics of U1 snRNPs towards the DSS_n_ and 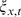 is the delta-correlated Gaussian white noise with 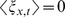 and 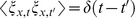. The movement of RNAPII along *y* is energetically driven via the hydrolysis of ATPs. As a result, the fluctuations in *y* are negligible and we use a deterministic description for RNAPII in **Eq. 1**.

Let 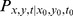 denote the joint probability of finding the snRNPs at position *x* and RNAPII at position *y* at time *t* given initial conditions *x_0_, y_0_*. The Fokker-Planck equation associated with the temporal evolution of 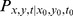 can be written as follows [31]:
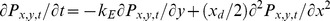
(2)Here the initial condition is 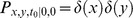, ensuring that at time *t_0_*, the probability of finding *x_0_* = 0, *y_0_* = 0 is normalized to one. The boundary conditions are as follows:
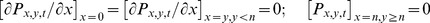
(2′)Here *x* = 0 as well as *x* = *y* (*y*<*n*) act as reflecting boundary conditions for the dynamics of snRNP. Whenever the snRNP tries to visit *x*≤0 or *x*≥*y* it is reflected back into *x* 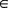
[*0, y*]. Here 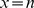 acts as absorbing boundary condition whenever 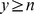.

Let 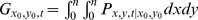 indicate the probability that RNAPII and snRNP are between position 0 and *n* at time *t* (given starting points *x_0_*, *y_0_*). Let 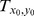 denote the mean first passage time (MFPT) associated with the binding of snRNP at DSS_n_ starting from initial conditions (*x_0_*, *y_0_*). From the definition of MFPT, 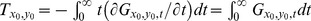. Noting that before time *n/k_E_*, the DSS_n_ has not emerged yet, we have:
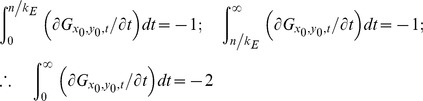
and therefore 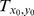 obeys the following backward type Fokker-Planck equation [31]:
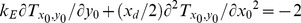
(3)with the following boundary conditions:
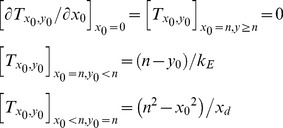
(3′)


**Figure 2:**
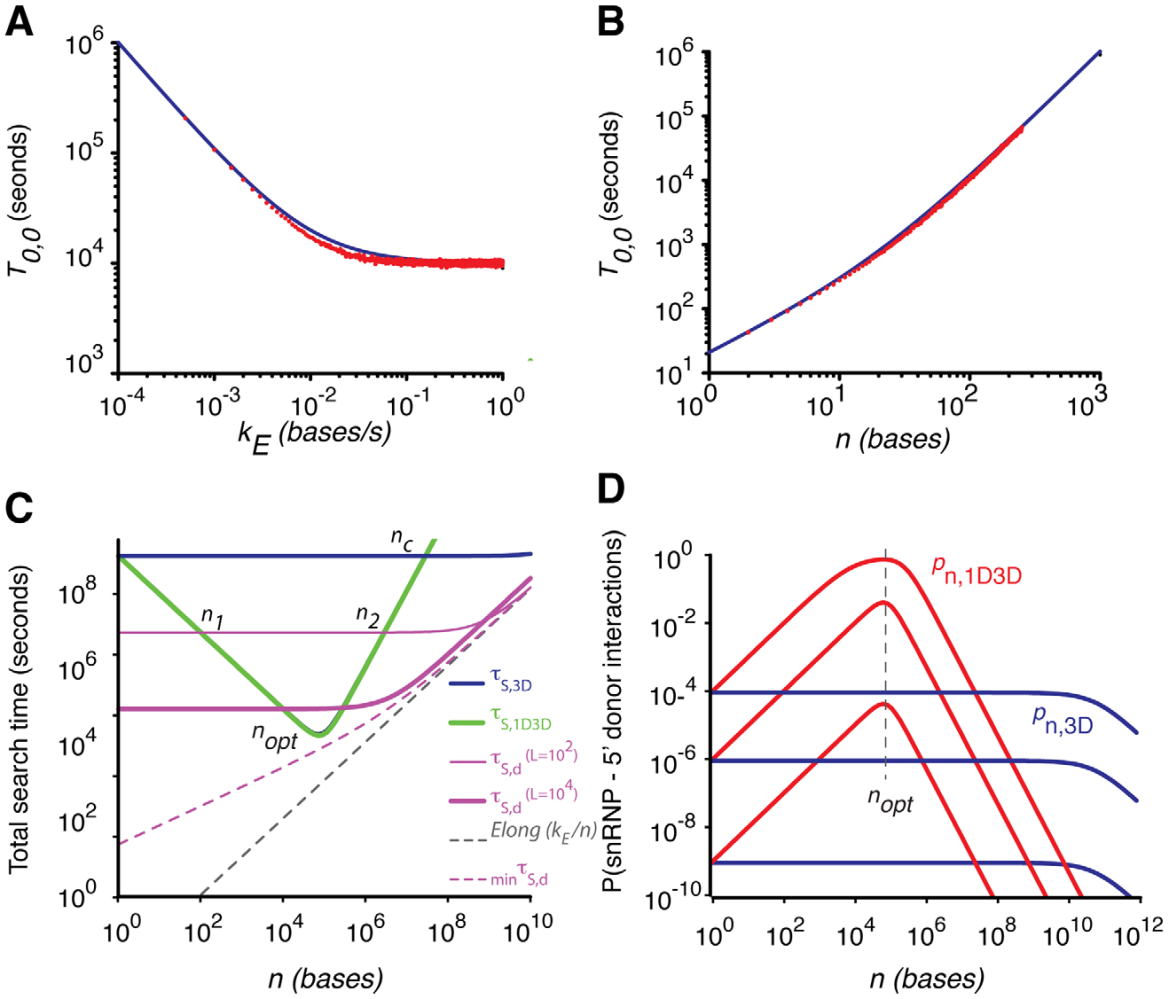
A–B. Validation of the expression for the mean first passage time (MFPT, in seconds) given by **Eq. 4** (blue) using random walk simulations (red) at different elongation rates *k_E_* (A) and different positions of the absorbing boundary *n* (B). Initial positions: *x_0_* = 0 (snRNP) and *y_0_* = 0 (RNAPII). *x_d_* = 1 bases^2^/s. In **A**, *n* = 100 bases and in **B**, *k_E_* = 1 base/s. Whenever the random walker (snRNP) hits the drifting reflecting boundary *x = y*, it is put back into the interval (0, *y*). Whenever *y = n* and *x = y* the random walker is removed from the system. The MFPT was calculated over 10^5^ random walk trajectories (Materials and Methods). **C.** Minimization of the overall search time by an snRNP to locate the splicing site DSS_n_ on the pre-mRNA when the search is via 3D only (*τ_S,3D_*, blue, **Eq. 7**), 1D+3D routes (*τ_S,1D3D_*, green, **Eq. 5**) or 1D+3D including snRNP dissociation (*τ_S,d_*, pink, **Eq. 9**, shown for two different values of the dissociation length L). There exists an optimum position of splice sites at around *n_opt_* = 4×10^4^ bases at which the 1D+3D search time is minimized. The time taken for a pure 3D search will be less than the combination of 1D and 3D search beyond *n_c_*∼2×10^7^ bases. The dashed black line indicates the transcription time (*k_E_/n*) and the pink dashed line indicates the minimum search time (*_min_τ_S,d_*, **Eq. 10**). Here the parameters are 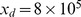 bases^2^/s, *k_E_ = 72* bases/s and 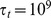 bases s. With a total of 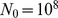 snRNPs and 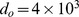 splicing-sites at a given active region of the nucleoplasm (∼1% of the total nascent pre-mRNAs) the search time scales down by a factor of (*d_0_/N_0_*). **D.** Variation of the overall probabilities associated with the interaction of snRNPs with DSS_n_ as a function of *n* for different snRNP concentrations (*N_0_* = 10^3^, 10^5^ and 10^7^ from bottom to top) (**Eqns 11** **–** **12**). The red curves show the probabilities including 1D and 3D search mechanisms (*p_n,1D3D_*, **Eq. 11**) and the blue curves show the probabilities including only 3D search mechanisms (*p_n,3D_*, **Eq. 12**). *p_n,1D3D_* reaches a maximum at the value *n_opt_*, which does not depend on *N_0_*. As *N_0_* increases, the optimum position of splicing sites on the pre-mRNA expands into a wider range of *n* values. Here the parameter settings were *k_off,n_ = 10* s^−1^ and other parameters as in part **C**.

We assume that the residence time associated with dissociation of the non-specific bound snRNPs from the pre-mRNA is much higher than the time required by the snRNPs to locate the 5′ donor splicing sites. As a result, we have introduced a reflecting boundary condition at *x* = 0 in the first boundary condition. The other boundary conditions can be directly derived from **Eq. 2′**. The second boundary condition describes the conditions where RNAPII transcription elongation is the limiting step and the third boundary condition describes the conditions where snRNP diffusion is the limiting step. The particular solution to **Eq. 3** for the boundary conditions in **Eqns 3′** can be written as follows: 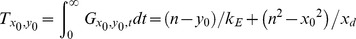
(4)Considering *x_0_* = 0 and *y_0_* = 0 (both RNAPII and snRNP start at the origin), we have 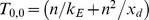. The first term is the time required to generate a pre-mRNA of *n* bases and the second term is the time required by the snRNPs to completely scan this pre-mRNA length via 1D diffusion. The validity of this equation for the MFPT under various values of *n* and *k_E_* is illustrated in **Figure 2A–B** using random walk simulations.

In line with site-specific DNA-protein interactions [27]–[30], we assume that snRNP molecules locate their respective DSS binding sites on the growing pre-mRNA via a combination of 1D and 3D diffusion-controlled collision routes. Under such conditions, from **Eq. 4** we find the average overall search time (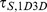) required by the snRNPs to locate DSS_n_ (*x_0_ = 0;y_0_ = 0*):
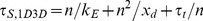
(5)


Here 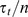 (units of seconds) is the 3D diffusion-controlled collision time required for non-specific binding of U1 snRNP with the pre-mRNA of length *n*. **Eq. 5** suggests that there exists an optimum position of DSS_n_ on the emerging pre-mRNA such that the search time required by the snRNPs to locate this DSS_n_ will be a minimum. This optimum value can be obtained by solving 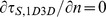 for *n*. The explicit real solution of the resulting cubic equation is:
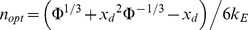
(6)where 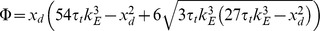. Upon substituting *n_opt_* in **Eq. 5** we find the minimum search time 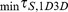.

In line with the prediction of the kinetic model, when the snRNPs locate the DSS_n_ via a purely 3D diffusion-controlled collision route, the overall search time is:
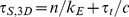
(7)In this equation, *c* (units of bases) is the sequence length within which the snRNPs can be captured at the 5′ donor site. A precise and tight binding would correspond to *c* = 1. Upon comparing this expression with **Eq. 5** we find that there exists a critical position on the pre-mRNA (*n_c_*) such that *τ_S,1D3D_ = τ_S,3D_*. Solving the cubic equation 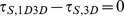 for *n* (**Figure 2C**):
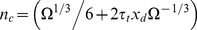
(8)where 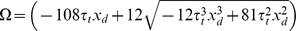.

While deriving **Eq. 5** we have assumed that the non-specific bound snRNP does not dissociate from the pre-mRNA chain until it reaches DSS_n_. We relax this assumption by modeling the search dynamics of snRNPs as multiple cycles of dissociation-scan-association events. In this modified version of the model, the non-specific bound snRNP can dissociate after scanning an average pre-mRNA length of *L* bases and then it re-associates back at the same or different location of the pre-mRNA chain. In this way, snRNPs are required to undergo at least (*n*/*L*) such association/dissociation events to scan the entire length of *n* bases. Under such conditions, the expression for the overall search time (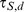) can be written as follows:
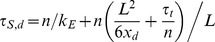
(9)Here *L^2^/6x_d_* is the average time required by the non-specific bound snRNPs to scan an average of *L* bases of pre-mRNA before the dissociation event. The scan length *L* depends on the magnitude of the interaction between the snRNPs and the pre-mRNA. When *L = n*, **Eq. 9** reduces to **Eq. 5**. When 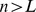, there exists an optimum value of *L* in **Eq. 9** at which 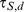 is a minimum: 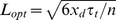. The corresponding minimum achievable search time is:
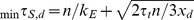
(10)


One should note that the optimum 1D scanning length can be achieved by the diffusing U1 snRNPs only when the inequality condition 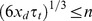 holds since by definition 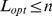. Further analysis shows that 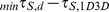 will reach a minimum only when 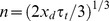. Upon comparing **Eqns 5** **,** **7** **and** **9** we find that when *n*<*n_c_*, then both 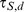 and 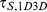 will be lower than 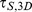. In the range *L* 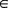
(0, *n_opt_*) the cubic equation 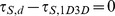 has two real solutions for *n* (*n_1_*∼*L* and *n_2_*, marked in **Figure 2C**) for *n*. When *n* 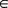
(*L*, *n_2_*), we find that 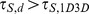. The relationship among these different search times is shown in **Figure 2C**. These results suggests that among the three possible modes of searching (pure 3D, 1D3D with multiple dissociations and 1D3D without dissociation), the 1D3D search mode of search without any dissociation event will be the most efficient and preferable one in the range *n* 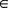
(*L*, *n_2_*) where *L* is the possible 1D scanning length associated with diffusion of U1 snRNPs along the emerging pre-mRNAs. We find from **Eqs. 9** **–** **10** that similar to the pure 3D diffusion mediated search time (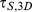), 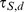 is also a monotonically increasing function of *n*. On the macroscopic level, the interactions of snRNPs with DSS_n_ can be described by the following chemical reaction scheme I:
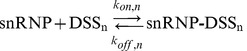
(Scheme I)Here 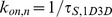 (bases^−1^s^−1^) is the bimolecular type forward on-rate constant associated with the site-specific interaction of snRNP with the DSS_n_ and 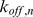 (s^−1^) is the respective dissociation or off-rate constant. The sequence of DSS_n_ plays critical role in determining the value of the off-rate. The number of snRNPs will be higher than the number of DSSs of a particular pre-mRNA transcript. In this situation, the thermodynamic probability of finding DSS_n_ (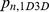) to be bound with snRNPs is:
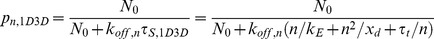
(11)Here *N_0_* is the total number of the freely diffusing snRNPs inside the nucleus. It follows from **Eqns 5** **–** **6** that the probability 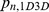 is maximized when *n = n_opt_* irrespective of the value of the intra nuclear concentrations of snRNPs or the amount of time for which the completely transcribed pre-mRNA chain stays inside the nuclear compartment for further post-transcriptional processing. On the other hand, when the snRNP search mode is purely via 3D routes then the probability (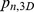) is a monotonically decreasing function of *n* (**Figure 2D**):
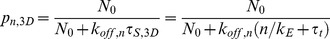
(12)From **Eqs 11** **–** **12**, we find 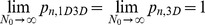 (all DSS_n_ bound by the snRNP given infinite concentration). Those splicing sites located closer to the optimum position (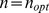) approach this limit faster. Using **Eq 11** we define the overall splicing efficiency of a transcript of length *n* as follows:
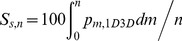
(13)


The value of the splicing efficiency 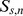 (between 0 and 100%) indicates how well exons present in a given pre-mRNA transcript of length *n* interact with the available pool of snRNPs, are subsequently spliced and hence get included in the final transcript. This means that the overall levels of the final transcript should be directly proportional to this splicing efficiency. There exists an optimum length of pre-mRNA transcript (*μ*) at which 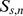 achieves a maximum. The optimum *μ* can be obtained by numerical solving 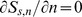 for *n*. The overall level of the final transcript will be maximum at 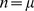 since the overall average probabilities associated with all those exons of the given pre-mRNA transcript of length *μ* to interact with the available snRNPs will be a maximum. We consider a transcript *c* of length *n* and its expression in tissue *k*. We define the overall signal as 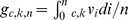 where 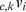 is the signal from the exon located at position *i* in transcript *c* in tissue *k*. With this definition we find that the maximum gene signal value of *n* occurs at 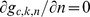 which means that when 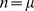 the equality 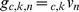 holds. This follows from the fact that 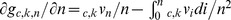.

### Comparison with experimental data

We compare the theoretical predictions outlined in the previous section with two different types of experimental measurements: (i) experiments based on exon microarray data and (ii) experiments based on high-throughput RNA sequencing data (RNAseq) (“Materials and Methods”). Upon substituting the parameters *τ_t_*, *k_E_* and *x_d_* into **Eq. 6** for the optimum position of the DSS on the pre-mRNA we find 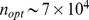 bases and the minimum achievable overall search time required by the snRNPs 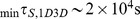. This search time is significantly higher than physiologically relevant timescales (for example, the cell's generation time). One should note that this higher timescale corresponds to the interaction of a single snRNP molecule with a single splicing site. The search time will be proportionately scaled up/down depending on the number of freely available snRNPs and nascent splicing sites inside the nucleus as 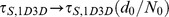. There are ∼2×10^4^ genes in the human genome, and there are on average ∼10 exons per gene. This means that there are *d_0_*∼4×10^3^ such splicing sites at any given active region of the chromosome (corresponding to ∼1% of the total pre-mRNAs being processed). With these values we find 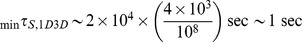. These results suggest that the appearance of the speckles where snRNPs are concentrated inside the nucleoplasm of higher eukaryotes is mainly to scale down the search time required by snRNPs to locate the splicing-sites on the pre-mRNA.

We conclude from the expression for the probability of finding the snRNP at position *n* (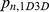, **Eq. 11**) that the DSS located at position 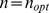 of the growing pre-mRNA will have more chances to interact with the available snRNPs. Here the minimization of the overall search time 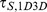 is achieved mainly via the enhancing effects of the increasing numbers of non-specific interactions of snRNPs with the growing pre-mRNA. We learn from **Eq. 8** that the inequality condition 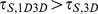 will hold whenever 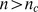. The current parameter settings yield 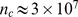 bases. Various single-cell studies using fluorescence recovery after photo bleaching (FRAP) provide an empirical estimate for the dissociation rate of snRNPs from the pre-mRNA chain: 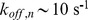 [24], [25], [26]. This is an overall off-rate that includes dissociation of snRNPs from both the non-specific and specific binding sites (the off-rate of snRNPs from the splicing sites will be lower than the off-rate from non-specific binding sites.) Using this value of 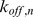, the limiting behavior of *p_n,1D3D_* and *p_n,3D_* as 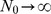 is demonstrated in **Figure 2D**. This figure suggests that the optimum position of DSS will spread into a wider range as the total concentration of snRNPs increases inside the nucleoplasm. Single molecule studies suggest an average 1D scanning length of *L*∼100 bases for the DNA-binding proteins under *in vivo* conditions [32]. With this value, upon solving the cubic equation 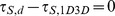 for *n* we find that *n_1_* = 100 and *n_2_* = 2×10^6^ bases. Since within this range 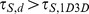, this result suggests that the dominating mode of searching of U1 snRNPs for the 5′ splicing sites is likely to be via the combination of 1D and 3D without dissociation for most of the pre-mRNAs.

**Figure 3:**
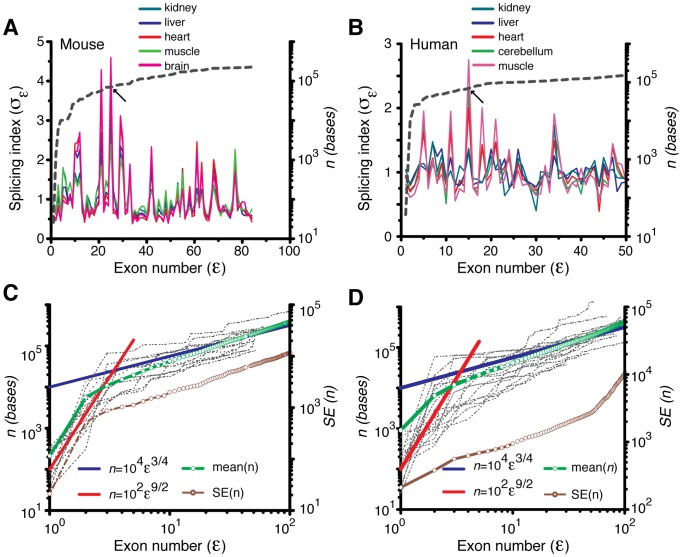
A. Example showing the splicing index (*σ_ε_*) as a function of the annotated exon number *ε* in mouse gene Dtnb (dystrobrevin beta, NM_007886, Affymetrix Transcript ID: 6792942). The example illustrates a constitutive splicing pattern across different tissues. The dashed line (right-axis) shows the exon position (bases) based on the annotations. The plot suggests that there is a coarse optimum exon position (arrow) associated with a maximum splicing index; across different genes this maximum is coarsely around the predicted value of *n*∼7×10^4^ bases in the original pre-mRNA. More examples are shown in **Figure S1**. **B.** Example showing the splicing index of the human vitrin gene (VIT, Affymetrix Transcript ID: 2477203, NM_053276). The format is the same as in part **A**. More examples are shown in **Figure S2**. **C–D.** Scaling relationship between exon number (*ε*) and exon position (*n*) on the pre-mRNA transcript for mouse (**C**) and human (**D**). Here positions versus exon numbers for 18 human genes (Transcript id (number of exons), 2598971 (93), 2975385(79), 3123036(30), 2688813(40), 2753440(153), 2975385(79), 2477073 (87), 2477203 (50), 2480700 (114), 2481308 (49), 2481379 (48), 2481929 (54), 2482505 (80), 2552368 (56), 2638509 (69), 2639734 (68), 2828564 (79), 2639552 (134) and 14 mouse genes (6991267 (39), 6946339 (86), 6770718 (40), 6839871 (51), 6946339 (86), 6998972 (64), 6990167 (147), 6805180 (61), 6805180 (61), 6747313 (25), 6747308 (23), 6747314 (38), 6751304 (96), 6771558 (18)) with different number of exons were obtained from the transcript and probe level Affymetrix annotations. In line with **Eq. 17**, when 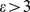 we approximate 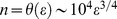. Green line-dots are the mean positions of exons. Brown line-dots are the standard error (SE) associated with the positions of exons. The scaling transformation 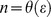 shows an error of ∼25%.

We considered microarray data evaluating exon levels in different tissues and species (Materials and Methods.) Examples of mouse and human constitutively spliced multi-exonic genes across various tissues are shown in **Figure 3A–B**. These examples, identified using the ranking metric defined in **Eq. 14**, suggest that there exists a broad optimum position of splicing sites on the pre-mRNA at which the probability associated with the inclusion of the associated exon is maximized. This position is approximately independent of the tissue analyzed. In these particular mouse and human genes (Dtnb dystrobrevin beta in mouse and VIT vitrin in human), this optimum exon number occurs at the pre-mRNA position of *n*∼5×10^4^ to 10^5^ bases (arrow in **Figure 3A–B**). Other examples are included in supplementary materials (**Figure S1, S2**). The position of the maximum splicing index value, independently of the tissue, occurs around *n_opt_*∼7×10^4^ bases as predicted by **Eq. 6**, with an error margin of ∼25%.

Overall analysis of the multi-exonic genes present in both human and mouse genomes revealed an average intron length of ∼4×10^3^ bases with a median of ∼10^3^ bases. Here the average length of exons is ∼2×10^2^ bases with a median of ∼10^2^ bases. Results of genome wide analysis of the median of exon positions on pre-mRNAs of human and mouse is shown in **Figure 3C–D** which reveals the following approximate scaling relationships between the positions (*n*) and the exon numbers (*ε*):
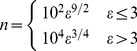
The standard error (SE) in such transformation is approximately 5 to 25% of the mean (*n*) for *ε* in the range 1 to 100 (**Figure 3C–D**). This suggests that the optimum positions *n_opt_* and _min_ *τ*_S,1D3D_ may be observed anywhere in the ±25% of the predicted values upon a genome wide averaging across exon numbers *ε*.

**Figure 4:**
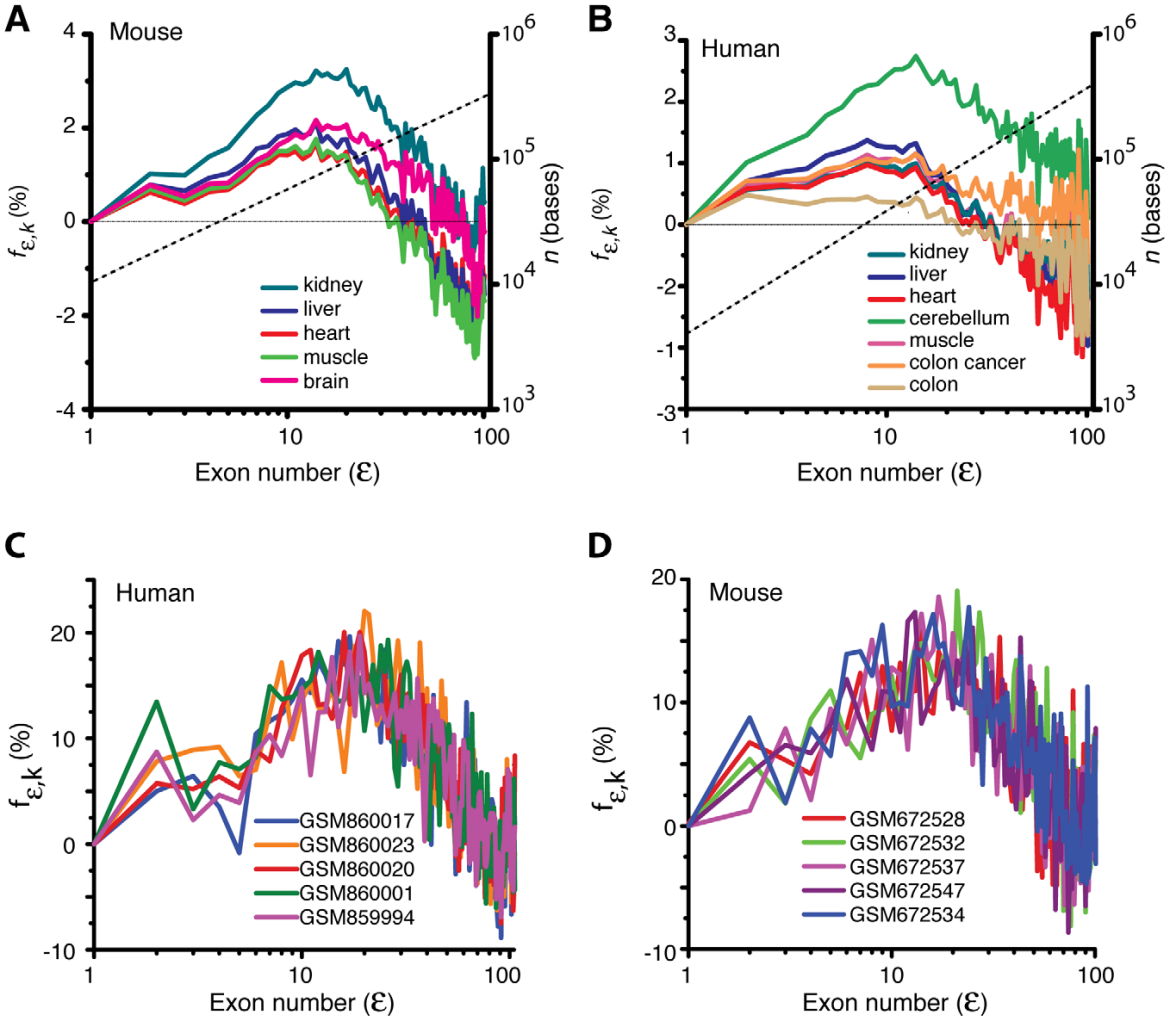
A–B. First exon normalized average signal for exon *ε* and tissue *k* (*f_ε,k_*, FENAS measured as defined in Eq. 15). Variation around these average signals is reported in **Figure S3**. The analyses are based on the exon microarray data for mouse (**A**) and human (**B**) derived from various tissues [33], [34] (Materials and Methods). Irrespective of the type of tissue, there exists an optimum exon number where the probability associated with that exon to be included in the final transcript is maximized. The dashed line shows the approximate average exon position in base pairs on the secondary axis. **C–D.** First exon normalized average signals (FENAS, **Eq. 15**) as a function of exon number *ε* for various cell types in mouse (**A**) and human (**B**). The data for this figure come from RNAseq experiments (Materials and Methods) (cf. parts A–B using microarray data).

The computed first exon normalized average signal (FENAS, defined in **Eq. 15**) associated with various mouse tissues (kidney, brain, liver, muscle and heart) and human tissues (cerebellum, kidney, liver, heart, muscle and normal and cancerous colon) is shown in **Figure 4A–B**. This figure indicates a maximum at approximately 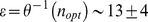. This value corresponds to the optimum position of the Affymetrix annotated exon on the pre-mRNA at 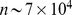 bases, which is broadly consistent with our theoretical predictions. We also compared the theoretical predictions with experimental data obtained from RNAseq experiments (Materials and Methods). The data from the exon level and transcript level signals obtained from RNASeq data of mouse brain and human T293 cells are shown in **Figure 4C–D**. The results from the RNASeq data are comparable to those from the microarray data and also reflect an optimum exon position, approximately around 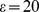.

**Figure 5:**
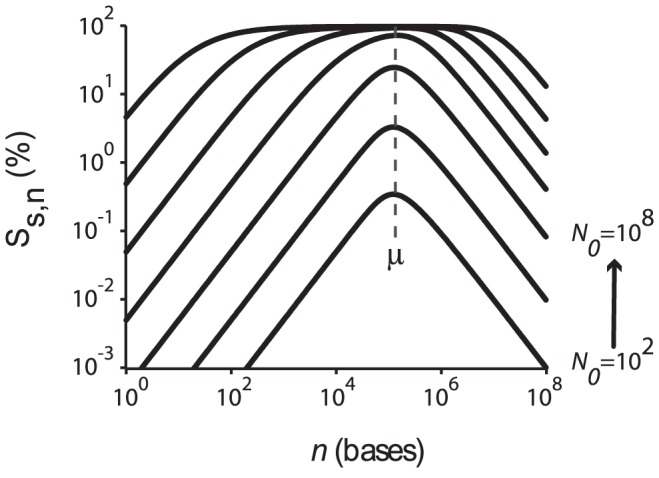
Overall splicing efficiency *S_s,n_* as a function of the transcript length *n* as defined in Eq. 13. The parameters are 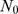 (10^2^, 10^3^, 10^4^, 10^5^, 10^6^, 10^7^ and 10^8^ molecules from bottom to top), 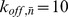 s^−1^, 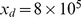 bases^2^ s^−1^, *k_E_ = 72* bases^1^s^−1^ and 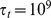 bases^1^ s^1^. At low *N_0_*, the splicing efficiency curve shows a maximum (*μ*) at a transcript length of 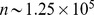 bases, corresponding to 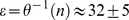 exons. As *N_0_* increases, the splicing efficiency will be almost >95% in the range of *n* values from 10^2^ to 10^6^.

**Figure 6:**
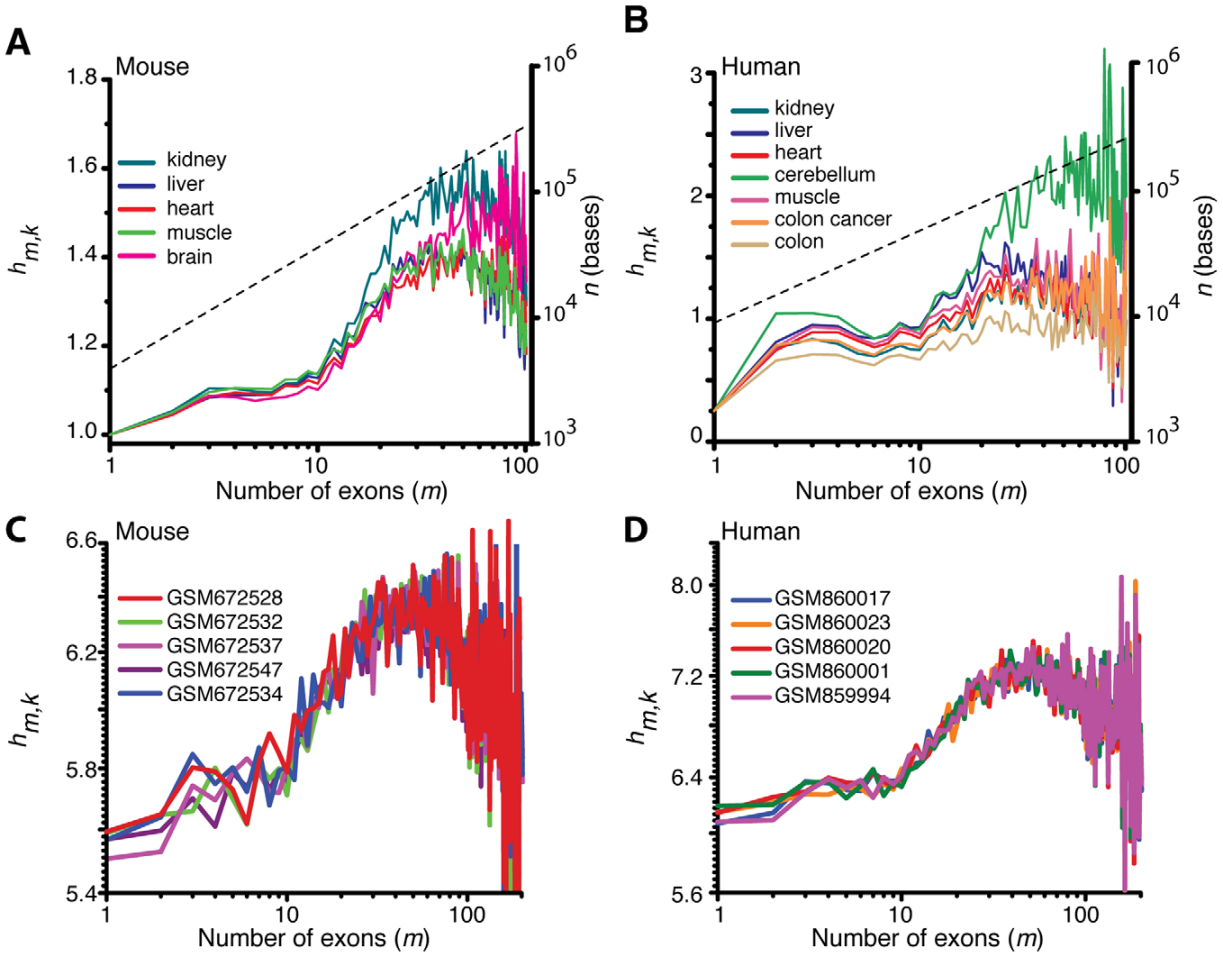
A–B. Genome-wide normalized average level of transcripts with *m* exons in the *k^th^* tissue (*h_m,k_*, Eq. 16) in mouse (A) and human (B). Variation around these average signals is reported in **Figure S4**. The data for this figure come from exon microarray experiments (Materials and Methods). These plots show a broad maximum approximately centered around *m*∼32 exons (arrow). The dashed line shows the approximate average exon position in base pairs on the secondary y axis. **C–D**. Genome-wide normalized average level of transcripts with *m* exons in the *k^th^* tissue (*h_m,k_*, **Eq. 16**) in human (**C**) and mouse (**D**). The data for this figure come from RNAseq experiments (Materials and Methods) (cf. data in **Figure 6** from microarray data).

Upon substituting 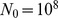 molecules, 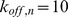 s^−1^ and the empirical values of *τ_t_*, *k_E_* and *x_d_* into **Eq. 13** and numerically solving it for the optimum transcript length *n = μ* we find 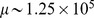 bases (**Figure 5**). This value corresponds to approximately 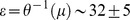 exons. From the theoretical analysis, we learn that the overall transcript signal of a given gene is maximized when the number of exons present in that gene is closer to this value. We find from **Figure 5** that the splicing efficiency is >95% whenever the length of the pre-mRNA transcript falls inside the range of ∼(10^2^–10^7^) bases. The distribution of transcript lengths both in humans and mouse is well within this broad range. Furthermore, we calculated the genome level averaged transcript signal across various mouse and human tissues using **Eq. 16**. **Figure 6** suggests that there is a broad maximum in the transcript signal approximately centered around 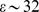 both based on the microarray data (**Figure 6A–B**) as well as the RNAseq data (**Figure 6C–D**). Within the expected error range of ±25%, these distributions and the location of the maxima are consistent with the theoretical predictions.

**Figure 7:**
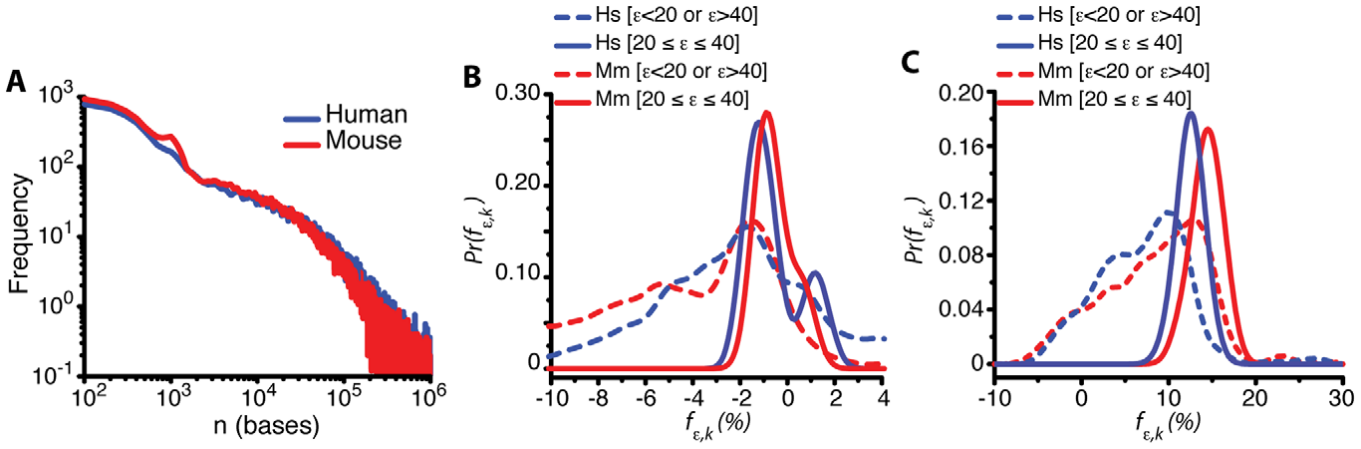
A. Distribution of transcript lengths based on the annotations (Materials and Methods). Mean values: 69900 bp (human) and 58300 bp (mouse); median values: 26209 bp (human) and 16972 bp (mouse). **B**. Distribution of FENAS values (*f_ε,k_* (%)) for human (red) and mouse (blue). The distributions are separately shown for those exon around the theoretically predicted optimum (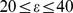, solid lines) or those exons that are far from *n_opt_* (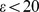 or 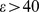, dashed lines). These distributions were constructed by considering all the values of *f_ε,k_* all the tissues (data pooled over *k*). The distribution of FENAS values for *ε* close to *n_opt_* was significantly different from the distribution of FENAS values for ε far from *n_opt_* both for human and mouse (t-test, *p*<0.05). **C**. Same as part **B** but using RNAseq data.

To further evaluate whether the experimental data are consistent with the existence of optimal exon positions, we computed the distribution of FENAS values for two separate broad ranges: (1) 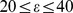 (i.e. around the theoretical optimum) and (2) 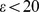 or 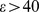 (i.e. far from the theoretical optimum). The distributions of FENAS signals were significantly different for these two ranges (t-test, p<0.05, **Figure 7**).

## Discussion

While the RNA polymerase II complex (RNAPII) is producing the pre-mRNA, multiple splicing factors diffuse inside the nucleus and initiate the recognition steps required in the process of splicing. Therefore, the ultimate mature mRNA product depends on several variables that affect the kinetics of these chemical and diffusion processes. These variables include RNAPII elongation speed and the presence of pausing events during transcription, the steric availability of splicing signals along the emerging pre-mRNA, exon and intron lengths, the abundance of different splicing factors and the sequence and hence affinity of those sequences for the splicing factors. Here we develop a simple theoretical framework that aims to capture the key interactions between transcriptional elongation and splicing.

The biophysical model proposed here can explain the effects of the stochastic search dynamics of small nuclear ribonucleo proteins (snRNPs) on the splicing pattern of eukaryotic genes. We considered two different ways to model the dynamics of snRNPs in the process of locating the splicing sites on the concomitantly evolving pre-mRNA: a pure three-dimensional diffusion process and a combination of three- and one-dimensional diffusion along the pre-mRNA. Our theoretical analysis on the coupled dynamics of transcription elongation and splicing revealed that there exists an optimum position of the splice sites on the growing pre-mRNA at which the time for snRNP binding is minimized (**Figure 2**). The minimization of the overall search-time is achieved mainly via increasing non-specific type interactions between the RNA binding domains of snRNPs and the pre-mRNA. The theory further revealed that there is an optimum transcript length that maximizes the sum of the probabilities for the exons in the transcript to interact with the snRNPs. This suggested that the overall transcript signal should be maximized at this transcript length.

We evaluated the theoretical predictions by analyzing exon microarray data from various mouse and human tissues (**Figures 3** **–** **6**). The empirical data revealed that the optimum position of the splice sites on the growing pre-mRNA occurs at ∼4.5×10^4^ bases and the optimum length of the transcript occurs at ∼7.5×10^4^ bases (corresponding approximately to the ∼11^th^ and ∼20^th^ exon in the genome wide first exon normalized average signal space.) The empirical data are broadly consistent with the theoretical predictions and the model captures, to a first approximation, some of the variability in exon level signals and splicing patterns.

Several computational algorithms have been developed to attempt to predict splicing patterns from DNA sequence. Most of the current splicing pattern prediction algorithms are solely based on *cis*-acting regulatory elements [21], [22], [23]. Typically each exon of a given pre-mRNA transcript is assigned a score depending on the presence or absence of exonic and intronic enhancer or silencer elements and their degree of conservation across different species [31]:. Using these exon level scores, transcript level scores are computed. Our work points out that, before computing the exonic scores for the presence of *cis*-acting elements, the ‘backbone’ of the scoring scheme assumes that all the exons are probabilistically equivalent. This uniform distribution of exon probabilities may hold only when the snRNP search mode is via pure 3D diffusion (**Figure 2D**) or the nuclear concentration of snRNPs is infinite. In more general scenarios, instead of a uniform distribution, our theoretical model suggests that the backbone of the scoring scheme should be given by the probability functional as defined in **Eq. 12** **–** **13**. In other words, the backbone of the scoring scheme is determined by the generic variables 2 (transcription elongation rate), 3 (interactions between RNAPII and snRNPs) and 4 (stochastic dynamics of snRNP search processes) as highlighted in the introduction. The model suggests that a modified scoring scheme would include the background model that accounts for the coupled kinetics of transcription and splicing in addition to the exonic scores for the presence of *cis*-acting regulatory elements.

The theoretical framework presented here provides initial steps to describe the coupled chemical and diffusion process that underlie transcription and splicing. While we focused here on generic variables that affect all transcripts and genes, a lot of the transcript-to-transcript and gene-to-gene variability depends on sequence specific factors, gene-specific transcription pausing events, regulation of transcriptional termination and the speed at which the mRNA is transported to the cytoplasm. The theory proposed here constitutes a starting point to build more sophisticated models that further incorporate important aspects of the biology that were not considered in this initial examination.

## Materials and Methods

### Datasets

To compare our theoretical predictions with experimental observations, we considered two different types of publicly available data: (i) exon microarray data and (ii) RNAseq data.

#### Exon microarray data

We analyzed mouse and human exon microarray data collected using Affymetrix arrays [33], [34]. We used exon level signal data collected in triplicate from five different mouse tissues (brain, kidney, muscle, liver and heart; mouse Mo-Ex 1.0) and five different human tissues (cerebellum, kidney, muscle, liver, heart; human Hu-Ex 1.0). We also considered the available sample microarray data from normal and cancerous human colon [33], [34].

#### RNAseq data

We analyzed BOWTIE generated RNASeq datasets [35], [36]. The data sets come from mouse brain (GSM672532, GSM672537, GSM672528, GSM672534 and GSM672547), and human 293T cells (GSM860026, GSM860020, GSM860017, GSM860001 and GSM9685994). The mouse annotations are based on the mm8 genome build and the human annotations are based on the hg18 genome build and the data were obtained from the GEO database [37], [38]. We used the information on sequence type annotation, sequence, and genomic alignment from the GEO files.

### Preprocessing of raw data

Experimental artifacts are introduced in the exon microarray data by factors such as cross-hybridizing probes, signal heterogeneity due to variation in the base composition of probes and signal variation due to fluctuations in the spot size of probes during microarray design. The cross-hybridization problem was solved by removing those probes showing hybridization at more than one location. Since the variations in probe level signals due to base composition, spot size and RT reaction are approximately random in nature, we assume that these errors are ameliorated by averaging over the scale normalized and background subtracted probe level signals of a probe set id, exon cluster id or transcript cluster id..

### Exon level analysis

Exon level signals are computed by averaging the probe-set id level signals contained in an exon-cluster id and transcript level signals are computed by averaging the exon level signals contained in a transcript cluster id. Only the Refseq annotated transcript cluster ids were considered for all the subsequent calculations. We used the standard Tukey biweight algorithm [39] to remove the outlier probe signals before computing the average. We considered multiple transcripts (indexed by *c*) and different tissues (indexed by *k*). Let *s_ε,c,k_* denote the log2 of the expression level of the *ε^th^* exon in transcript number *c* and tissue number *k*. The relative probability 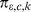 associated with the *ε* ^th^ exon to get included in the final transcript was defined as 
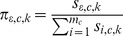
 where *m_c_* is the total number of exons in transcript *c*. The probability 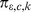 is directly related to the splicing-index (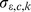) of the associated exon which is a measure of the extent of alternative splicing in that transcript, defined as 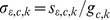 where *g_c,k_* is the overall level of transcript *c* in tissue *k*. In addition to the stochastic component, other splicing variables such as the presence of *cis*-acting regulatory elements including splicing enhancers and suppressors can significantly modify the probabilities defined here.

To evaluate the expression derived in **Eqns (11** **–** **12)** we need a splicing probability profile of a pre-mRNA transcript that contains multiple exons spliced in a ‘constitutive’ manner across various tissues. Here we use the term ‘constitutive splicing’ to indicate the splicing pattern of a given pre-mRNA that is conserved across various tissues in a given organism. We use the following variance-based scoring metric to rank and select such constitutive transcripts from the pool of multi-exonic pre-mRNAs of a given genome:
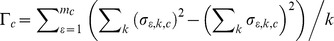
(14)We ranked the transcripts based on 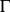 and we considered the top 25 transcripts to evaluate the theoretical predictions (these 25 transcripts represent the ones with minimal variation in the splicing index across different tissues as defined by the index 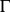). For a single-exon transcript, 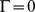. Earlier studies show that the majority of multi-exonic pre-mRNAs are spliced alternatively [21], [23]. This suggests that the number of constitutively spliced examples available to evaluate our model is limited.

We assume that the effects of *cis*-acting elements associated with a given exon number of various genes across the genome is approximately a symmetric random variable. That is, we assume that both the *cis*-acting enhancers as well as silencer elements are found on the genome with equal probabilities. Under this assumption, we expect that averaging over the first exon normalized signals (FENAS) of a given exon number across all the available multi exonic genes in the entire genome of an organism will essentially reduce up- and down-regulatory effects of the *cis*-acting elements apart from a local normalization of the exon signals within a gene. While carrying out this averaging process, the start and stop positions of each *ε* ^th^ exon of the pre-mRNA of different gene transcripts is also averaged out in such a way that in the overall averaged signal space the exons of average length are equally separated or flanked by the average length of introns of the genome. We define the FENAS metric as follows:
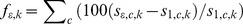
(15)Here 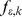 is the genome level FENAS (±%) of the *ε* ^th^ exon in tissue *k*. To compare **Eq. (15)** with **Eqns (11** **–** **12)**, we use the genome-wide scaling 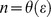, that is, the position of DSS_n_ is a function of the exon number *ε* (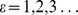). We note that 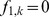 and 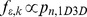. To evaluate **Eq. (11** **–** **12)**, the average signals associated with the final transcripts with various numbers of exons at the genome level were calculated as follows:
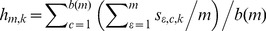
(16)Here 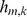 is the genome level average signal of those transcripts with *m* exons in the *k* ^th^ tissue; *b(m)* is the total number of transcripts with *m* exons.

### Analysis of RNASeq data

Exon microarrays possess very few probe sets per exon cluster id. Therefore, we also analyzed the number of sequence reads from RNASeq data (see datasets above). For this purpose we considered the start and end position of each transcript and exon and summed over the number of reads from RNASeq data. These signal profiles were used to compute the first exon normalized average signals FENAS as described in **Eqn 15**. To compute the transcript level signal we considered the start and stop position of each transcript and summed over the number of reads from RNASeq data within this range.

### Parameter estimation from experimental data

In order to compare the theoretical predictions with experimental measurements we estimate the kinetic and diffusion parameters required to quantitatively evaluate the theoretical equations from experimental studies. Single molecule data from the human U2OS osteosarcoma cell line shows an *in vivo* transcription elongation rate for RNAPII of 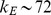 bases s^−1^ [40]. Single cell studies on BAC HeLa and E3 U2OS cell lines suggest that the overall diffusion coefficient for the U1-70K snRNP inside the nuclear splicing region is on the order of 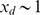 µm^2^/s (∼8×10^6^ bases^−2^s^−1^) [24], [25], [26]. This value is close to the 3D diffusion coefficient associated with the dynamics of protein molecules inside the cytoplasm of prokaryotic systems [32]. The 1D diffusion coefficient associated with the diffusion dynamics of snRNPs on the pre-mRNA chain is not clearly known. Single molecule studies in *E. coli* [40] showed a numerical value of 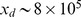 bases^2^s (∼0.092 µm^2^/s) for the 1D diffusion coefficient associated with the dynamics of transcription factors along the DNA. This value is approximately 10 times smaller than the experimentally observed overall diffusion coefficient of U1 snRNP inside the nucleus. The experimentally observed fast diffusion coefficient can be attributed to the more flexible nature of single stranded pre-mRNAs compared to the double stranded DNA chain. The nuclear diameter of a typical human cell is ∼6 µm and the corresponding volume will be ∼10^−16^ m^3^. The concentration of a single snRNP molecule or its single DSS binding site on the pre-mRNA in this volume will be ∼20 pM. When the length of the pre-mRNA is *n* bases, there should be at least ∼*n* non-specific binding sites for snRNPs. Single cell experimental studies suggested the timescale required by the snRNPs to non-specifically interact with the pre-mRNA is about ∼0.1 s [24], [25], [26]. This value suggests an overall off-rate 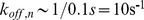. There are approximately *N_0_*∼10^8^ snRNPs inside the nuclear volume [41] which means that the number of non-specific collisions that can happen between a single snRNP molecule and the growing pre-mRNA chain will be in the order of 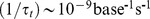.

## Supporting Information

Figure S1 **Mouse.** This supplementary figure provides further examples showing the splicing index as a function of the annotated exon number (the format is the same as the one in **Figure 3A**; see **Figure 3A** caption for details). **A.** Affymetrix Transcript ID: 6747308 Gene: Lypla1, lysophospholipase 1, NM_008866 **B.** Affymetrix Transcript ID: 6865573 Gene: Cep120, centrosomal protein 120, NM_178686 **C.** Affymetrix Transcript ID: 6770693 Gene: Osbpl8, oxysterol binding protein-like 8, NM_175489 **D.** Affymetrix Transcript ID: 6770718 Gene: Nap1l1, nucleosome assembly protein 1-like 1 NM_015781 **E.** Affymetrix Transcript ID: 6839871 Gene: Hira, histone cell cycle regulation defective homolog A, NM_010435. **F.** Affymetrix Transcript ID: 6814200 Gene: Mus musculus mRNA for mKIAA0947 protein. ENSMUST00000043493//ENSEMBL//hypothetical protein LOC218333 isoform 1 gene: ENSMUSG00000034525 **G.** Affymetrix Transcript ID: 6915559 Gene: Fggy, FGGY carbohydrate kinase domain containing, NM_029347 **H.** Affymetrix Transcript ID: 6825511 Gene: NM_028032, Ppp2r2a, protein phosphatase 2 (formerly 2A) regulatory subunit B (PR 52) alpha isoform.
(PDF)

Figure S2 **Human.** This supplementary figure provides further examples showing the splicing index as a function of the annotated exon number (the format is the same as the one in **Figure 3B**; see **Figure 3B** caption for details). **A.** Affymetrix Transcript ID: 2477073, NM_016441, CRIM1, cysteine rich transmembrane BMP regulator 1 (chordin-like). B. Affymetrix Transcript ID: 2481379, NM_172311, STON1-GTF2A1L, STON1-GTF2A1L read through transcript. **C.** Affymetrix Transcript ID: 2482505, NM_003128, SPTBN1, spectrin beta, non-erythrocytic 1. **D.** Affymetrix Transcript ID: 2639552, NM_003947//KALRN//kalirin, RhoGEF kinase. **E.** Affymetrix Transcript ID: 2639734, NM_007064//KALRN//kalirin, RhoGEF kinase. **F.** Affymetrix Transcript ID: 2829171, NM_003202//TCF7//transcription factor 7 (T-cell specific, HMG-box). **G.** Affymetrix Transcript ID: 3179975, NM_005392//PHF2//PHD finger protein 2. **H.** Affymetrix Transcript ID: 3183604, NM_021224//ZNF462//zinc finger protein 462.
(PDF)

Figure S3 **This figure provides complementary data to** **Figure 4** **.** **A–B.** Standard error of the FENAS signal for mouse (**A**) and human (**B**). There is one line for each tissue but the curves overlap. **C–D**. Number of transcripts (count) with a given exon number for mouse (**C**) and human (**D**).
(PDF)

Figure S4 **This figure provides complementary data to** **Figure 6** **.** **A–B.** Standard error of *h_m,k_* for mouse (**A**) and human (**B**). **C–D**. Number of transcripts (count) with a given number of exons in mouse (**C**) and human (**D**).
(PDF)

Text S1 **List of variables defined in the text.**
(PDF)
