# Supplementary material for: Theory on the Coupled Stochastic Dynamics of Transcription and Splice-Site Recognition
Source: PLoS Comput Biol. 2012 Nov 1;8(11):e1002747. doi: 10.1371/journal.pcbi.1002747 (PMC3486868; doi:10.1371/journal.pcbi.1002747)
Supplement: Figure S1 — Mouse. This supplementary figure provides further examples showing the splicing index as a function of the annotated exon number (the format is the same as the one in Figure 3A ; see Figure 3A caption for details). A. Affymetrix Transcript ID: 6747308 Gene: Lypla1, lysophospholipase 1, NM_008866 B. Affymetrix Transcript ID: 6865573 Gene: Cep120, centrosomal protein 120, NM_178686 C. Affymetrix Transcript ID: 6770693 Gene: Osbpl8, oxysterol binding protein-like 8, NM_175489 D. Affymetrix Transcript ID: 6770718 Gene: Nap1l1, nucleosome assembly protein 1-like 1 NM_015781 E. Affymetrix Transcript ID: 6839871 Gene: Hira, histone cell cycle regulation defective homolog A, NM_010435. F. Affymetrix Transcript ID: 6814200 Gene: Mus musculus mRNA for mKIAA0947 protein. ENSMUST00000043493//ENSEMBL//hypothetical protein LOC218333 isoform 1 gene: ENSMUSG00000034525 G. Affymetrix Transcript ID: 6915559 Gene: Fggy, FGGY carbohydrate kinase domain containing, NM_029347 H. Affymetrix Transcript ID: 6825511 Gene: NM_028032, Ppp2r2a, protein phosphatase 2 (formerly 2A) regulatory subunit B (PR 52) alpha isoform. (PDF) [file pcbi.1002747.s001.pdf]

Figure S1

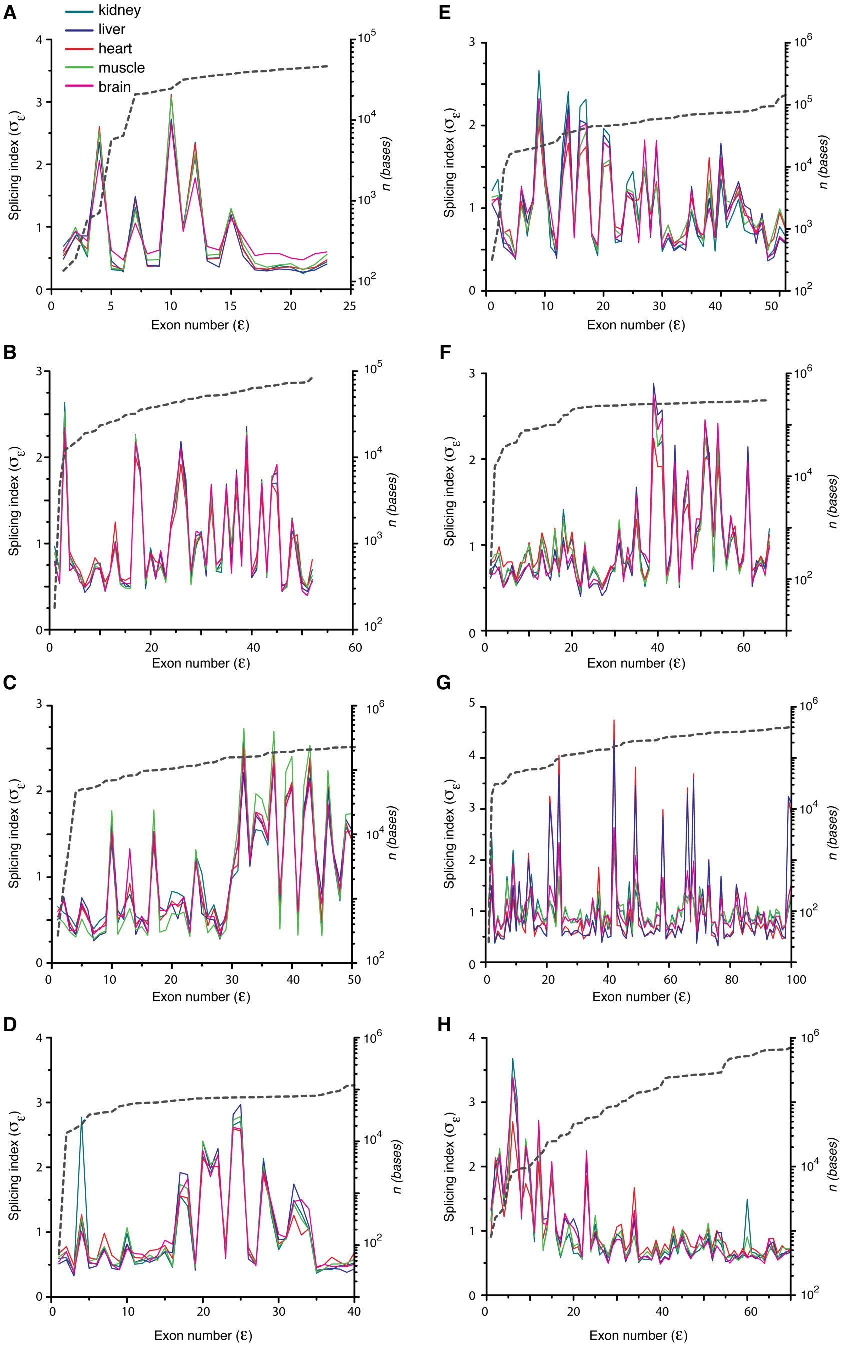

This supplementary figure provides further examples showing the splicing index as a function of the annotated exon number (the format is the same as the one in Figure 3A; see Figure 3A caption for details).

A. Affymetrix Transcript ID: 6747308 Gene: Lypla1, lysophospholipase 1, NM_008866

B. Affymetrix Transcript ID: 6865573 Gene: Cep120, centrosomal protein 120, NM_178686

C. Affymetrix Transcript ID: 6770693 Gene: Osbpl8, oxysterol binding protein-like 8, NM_175489

D. Affymetrix Transcript ID: 6770718 Gene: Nap1l1, nucleosome assembly protein 1-like 1 NM_015781

E. Affymetrix Transcript ID: 6839871 Gene: Hira, histone cell cycle regulation defective homolog A, NM_010435.

F. Affymetrix Transcript ID: 6814200 Gene: Mus musculus mRNA for mKIAA0947 protein. ENSMUST00000043493 // ENSEMBL // hypothetical protein LOC218333 isoform 1 gene: ENSMUSG00000034525

G. Affymetrix Transcript ID: 6915559 Gene: Fggy, FGGY carbohydrate kinase domain containing, NM_029347

H. Affymetrix Transcript ID: 6825511 Gene: NM_028032, Ppp2r2a, protein phosphatase 2 (formerly 2A) regulatory subunit B (PR 52) alpha isoform
