# Supplementary material for: Theory on the Coupled Stochastic Dynamics of Transcription and Splice-Site Recognition
Source: PLoS Comput Biol. 2012 Nov 1;8(11):e1002747. doi: 10.1371/journal.pcbi.1002747 (PMC3486868; doi:10.1371/journal.pcbi.1002747)
Supplement: Figure S2 — Human. This supplementary figure provides further examples showing the splicing index as a function of the annotated exon number (the format is the same as the one in Figure 3B ; see Figure 3B caption for details). A. Affymetrix Transcript ID: 2477073, NM_016441, CRIM1, cysteine rich transmembrane BMP regulator 1 (chordin-like). B. Affymetrix Transcript ID: 2481379, NM_172311, STON1-GTF2A1L, STON1-GTF2A1L read through transcript. C. Affymetrix Transcript ID: 2482505, NM_003128, SPTBN1, spectrin beta, non-erythrocytic 1. D. Affymetrix Transcript ID: 2639552, NM_003947//KALRN//kalirin, RhoGEF kinase. E. Affymetrix Transcript ID: 2639734, NM_007064//KALRN//kalirin, RhoGEF kinase. F. Affymetrix Transcript ID: 2829171, NM_003202//TCF7//transcription factor 7 (T-cell specific, HMG-box). G. Affymetrix Transcript ID: 3179975, NM_005392//PHF2//PHD finger protein 2. H. Affymetrix Transcript ID: 3183604, NM_021224//ZNF462//zinc finger protein 462. (PDF) [file pcbi.1002747.s002.pdf]

Figure S2

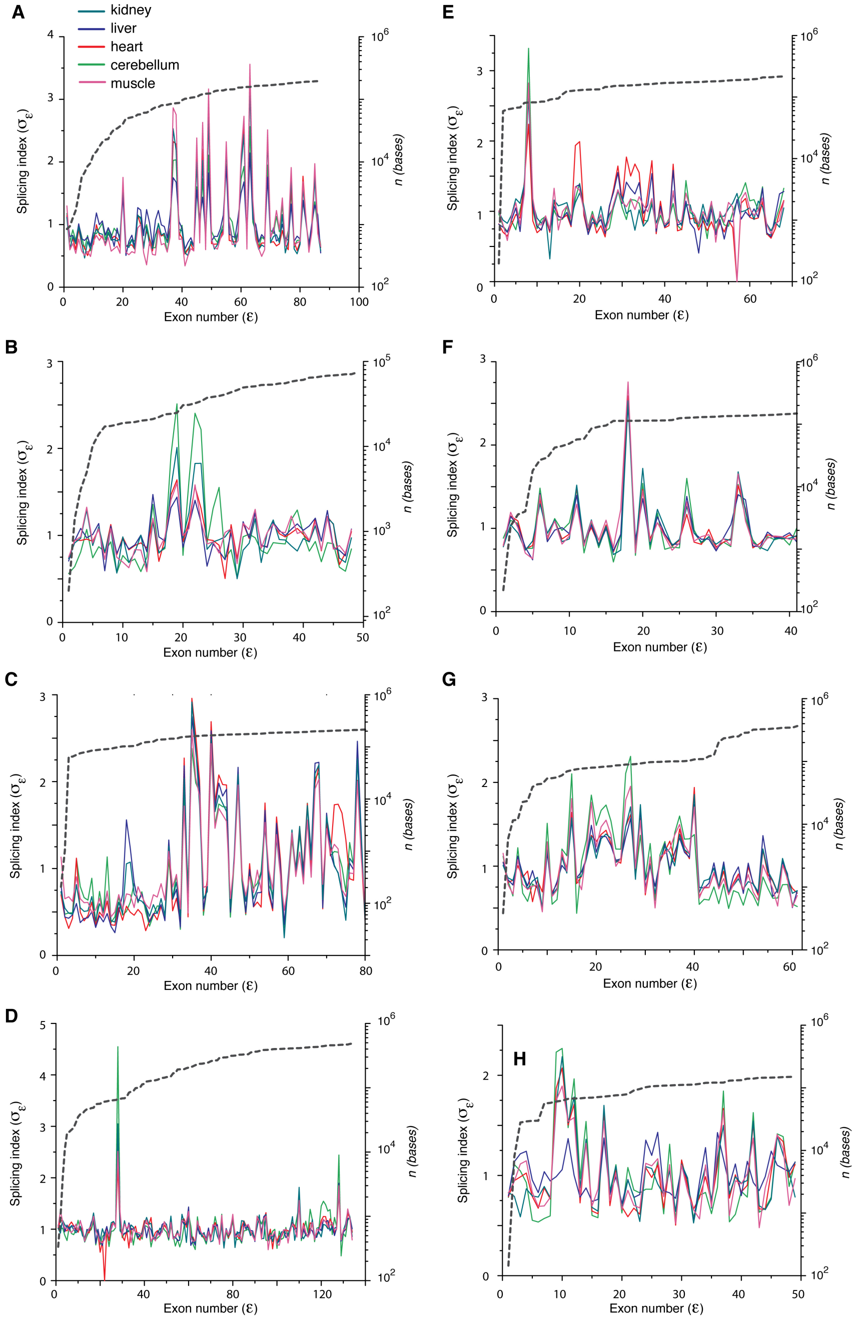

This supplementary figure provides further examples showing the splicing index as a function of the annotated exon number (the format is the same as the one in Figure 3B; see Figure 3B caption for details).

A. Affymetrix Transcript ID: 2477073, NM_016441, CRIM1, cysteine rich transmembrane BMP regulator 1 (chordin-like).

B. Affymetrix Transcript ID: 2481379, NM_172311, STON1-GTF2A1L, STON1-GTF2A1L read through transcript.

C. Affymetrix Transcript ID: 2482505, NM_003128, SPTBN1, spectrin beta, non-erythrocytic 1.

D. Affymetrix Transcript ID: 2639552, NM_003947 // KALRN // kalirin, RhoGEF kinase.

E. Affymetrix Transcript ID: 2639734, NM_007064 // KALRN // kalirin, RhoGEF kinase.

F. Affymetrix Transcript ID: 2829171, NM_003202 // TCF7 // transcription factor 7 (T-cell specific, HMG-box).

G. Affymetrix Transcript ID: 3179975, NM_005392 // PHF2 // PHD finger protein 2.

H. Affymetrix Transcript ID: 3183604, NM_021224 // ZNF462 // zinc finger protein 462.
