# Supplementary material for: Theory on the Coupled Stochastic Dynamics of Transcription and Splice-Site Recognition
Source: PLoS Comput Biol. 2012 Nov 1;8(11):e1002747. doi: 10.1371/journal.pcbi.1002747 (PMC3486868; doi:10.1371/journal.pcbi.1002747)
Supplement: Figure S3 — This figure provides complementary data to Figure 4 . A–B. Standard error of the FENAS signal for mouse (A) and human (B). There is one line for each tissue but the curves overlap. C–D. Number of transcripts (count) with a given exon number for mouse (C) and human (D). (PDF) [file pcbi.1002747.s003.pdf]

Figure S3

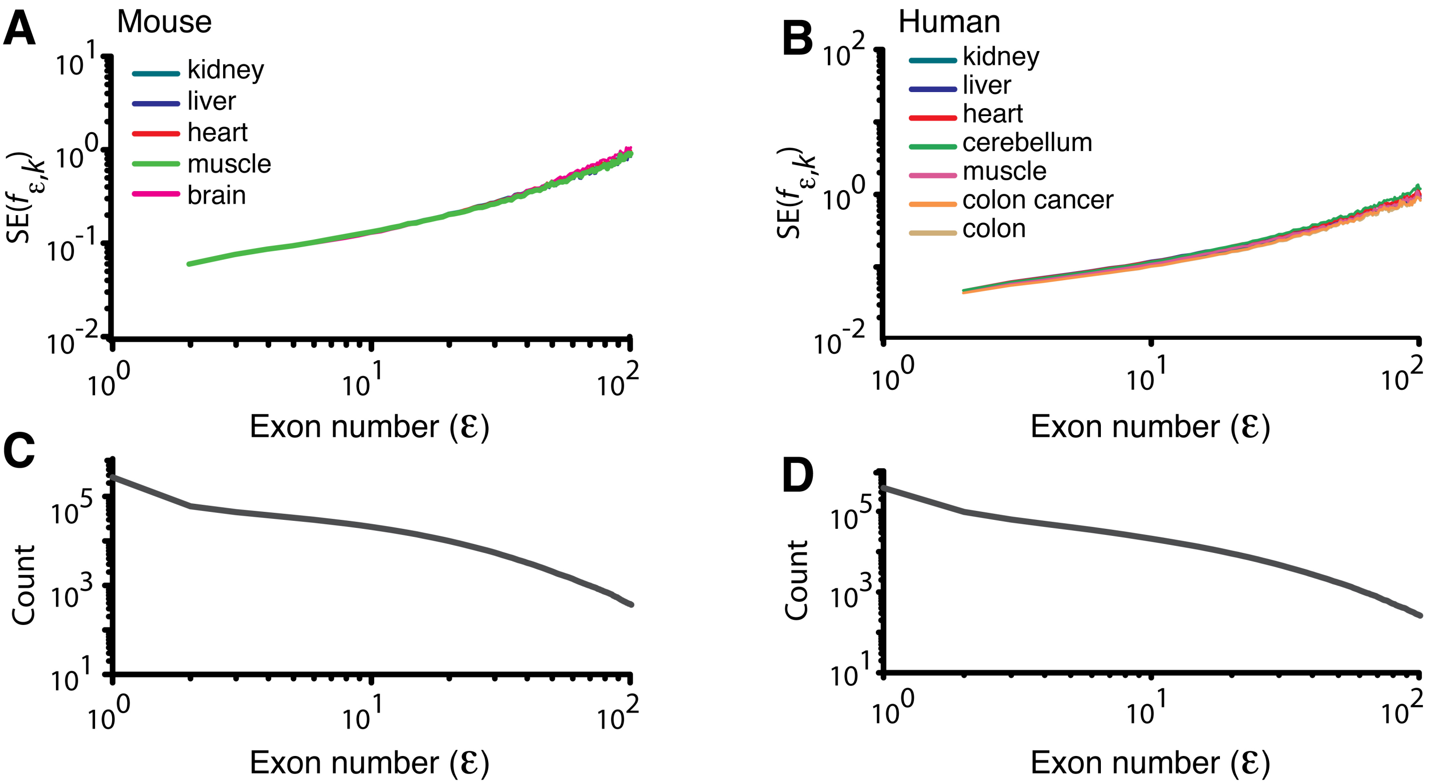

This figure provides complementary data to Figure 4.

A-B. Standard error of the FENAS signal for mouse (A) and human (B). There is one line for each tissue but the curves overlap.

C-D. Number of transcripts (count) with a given exon number for mouse (C) and human (D).
