# Supplementary material for: Theory on the Coupled Stochastic Dynamics of Transcription and Splice-Site Recognition
Source: PLoS Comput Biol. 2012 Nov 1;8(11):e1002747. doi: 10.1371/journal.pcbi.1002747 (PMC3486868; doi:10.1371/journal.pcbi.1002747)
Supplement: Figure S4 — This figure provides complementary data to Figure 6 . A–B. Standard error of hm,k for mouse (A) and human (B). C–D. Number of transcripts (count) with a given number of exons in mouse (C) and human (D). (PDF) [file pcbi.1002747.s004.pdf]

Figure S4

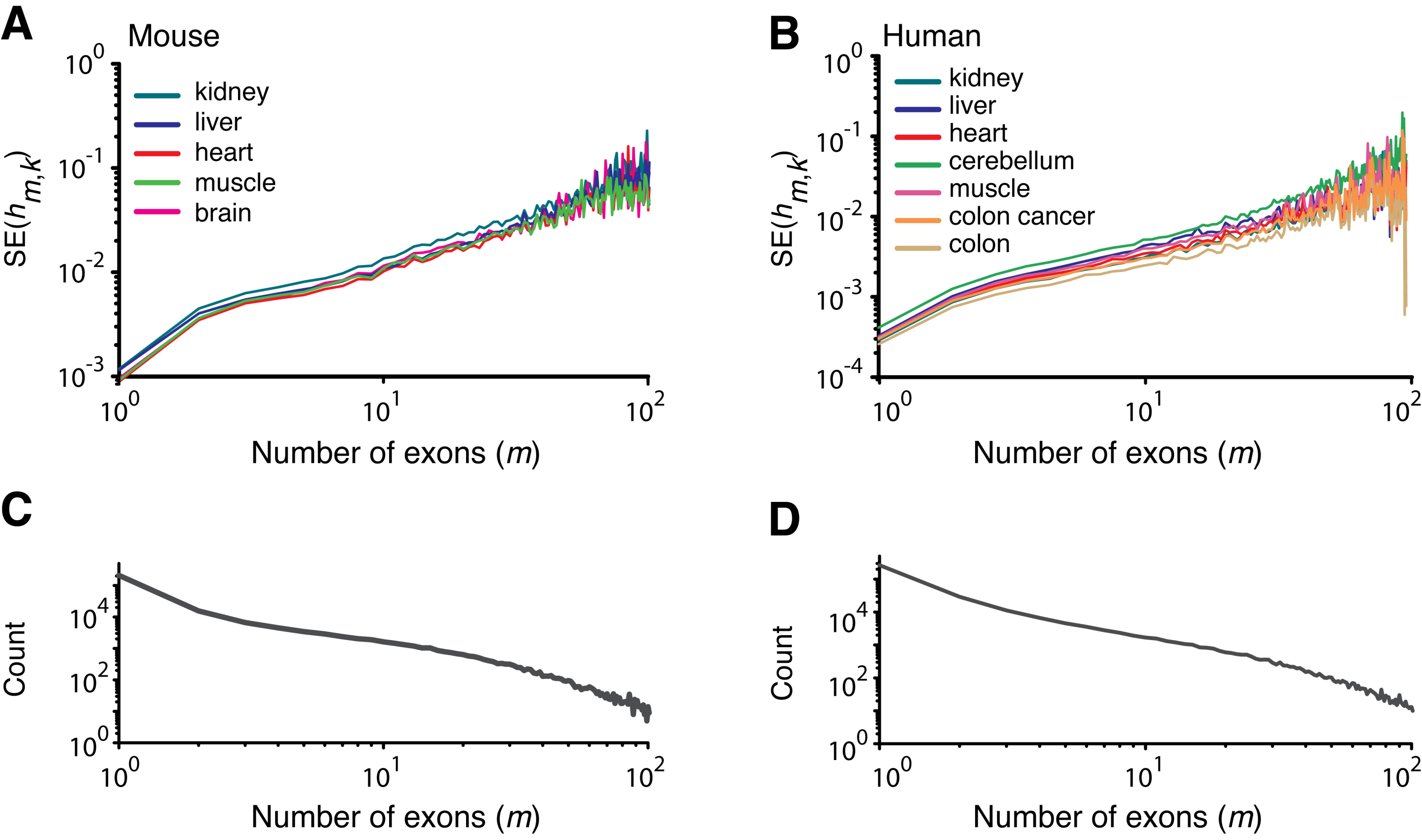

This figure provides complementary data to Figure 6.
A-B. Standard error of $h_{m,k}$ for mouse (A) and human (B).
C-D. Number of transcripts (count) with a given number of exons in mouse (C) and human (D).
