# Supplementary material for: Theory on the Coupled Stochastic Dynamics of Transcription and Splice-Site Recognition
Source: PLoS Comput Biol. 2012 Nov 1;8(11):e1002747. doi: 10.1371/journal.pcbi.1002747 (PMC3486868; doi:10.1371/journal.pcbi.1002747)
Supplement: Text S1 — List of variables defined in the text. (PDF) [file pcbi.1002747.s005.pdf]

Variables in Murugan and Kreiman – A theoretical framework of transcription-splicing coupling

Variable	Units	Definition	Description	Where defined	Where used	Comment
x	bases		snRNP position	**Page 4**	**F1, Eq1-4**	$x(t=0)=x_0$
y	bases		RNAPII position	**Page 4**	**F1, Eq1-4**	$y(t=0)=y_0$
t	sec		Time	**Page 4**	**F1, Eq1-4**	
x_d	bases^2/sec		1D diffusion constant for snRNP	**Eq1**	**F1, Eq1-12**	0.092 μm^2/sec ~ 8x10^5 bases^2/s
k_E	bases/sec		RNAPII elongation rate	**Eq1**	**F1, F2, Eq3-13**	72 bases/sec
$\xi_{x,t}$			Gaussian white noise	**Eq1**	**Eq1**	
n	bases		position of IEJ	**Page 4**	**F1, F2, Eq4-7**	
$P_{x,y,t}$			Joint probability	**Eq2**	**Eq2**	
$G_{x_0,y_0,t}$		$G_{x_0,y_0,t} = \int_0^n \int_0^n P_{x,y,t\|x_0,y_0,0}\, dx\, dy$	Probability that *x* and *y* are between 0 and *n*	**Page 5 (Eq3)**	**Eq3**	
T_{x_0,y_0}	Sec	$T_{x_0,y_0} = -\int_0^\infty t\partial_t G_{x_0,y_0,t} dt = \int_0^\infty G_{x_0,y_0,t} dt$	Mean first passage time	**Page 5 (Eq4)**	**F2A,B Eq4**	
$\tau_{S,1D3D}$	Sec	$\tau_{S,1D3D} = n/k_E + n^2/x_d + \tau_t/n$	Average overall search time (including 1D and 3D)	**Eq5**	**F2C Eq5**	
τ_t	bases sec		3D diffusion-controlled time constant	**Eq5**	**Eq5-11**	10^9 bases s
n_{opt}	bases	$n_{opt} = \left(\Phi^{1/3} + x_d^2\Phi^{-1/3} - x_d\right)/6k_E$	Solution when minimizing **Eq5**	**Eq6**	**F2, F4 Eq56**	
Φ		$\Phi = x_d\left(54\tau_t k_E^3 - x_d^2 + 6\sqrt{3\tau_t k_E^3\left(27\tau_t k_E^3 - x_d^2\right)}\right)$		**Eq6**	**Eq6**	
$_{\min}\tau_{S,1D3D}$	Sec		Using n_{opt} in **eq5**	**Page 6**	**F2C**	
$\tau_{S,3D}$	Sec	$\tau_{S,3D} = n/k_E + \tau_t$	Average overall search time (3D only)	**Page 6 (Eq7)**	**F2C Eq7**	
n_c	Bases	$n_c = \left(\Omega^{1/3}/6 + 2\tau_t x_d \Omega^{-1/3}\right)$	Position where $\tau_{S,1D} = \tau_{S,1D3D}$	**Eq8**	**F2C**	
Ω		$\Omega = \left(-108\tau_t x_d + 12\sqrt{-12\tau_t^3 x_d^3 + 81\tau_t^2 x_d^2}\right)$		**Eq8**	**Eq8**	
L	Bases		Average dissociation length	**Before Eq9**	**Eq9**	
$\tau_{S,d}$	Sec	$\tau_{S,d} = n/k_E + n\left(L^2/(6x_d) + \tau_t/n\right)/L$	Search time with snRNP dissociation	**Eq9**	**F2C Eq9**	
L_{opt}	Bases	$L_{opt} = \sqrt{6x_d\tau_t/n}$	Minimizes $\tau_{S,d}$	**After Eq9**		
$_{\min}\tau_{S,d}$	Sec	$_{\min}\tau_{S,d} = n/k_E + \sqrt{2\tau_t n/3x_d}$	Using L_{opt} in **Eq9**	**Eq10**	**Eq10**	
$k_{on,n}$	bases^{-1} sec^{-1}	$k_{on,n} = 1/\tau_{S,1D3D}$	Bimolecular forward on-rate constant	Scheme I		
$k_{off,n}$	sec^{-1}		Dissociation constant	Scheme I	**Eq11**	10 s^{-1}

Variables in Murugan and Kreiman – A theoretical framework of transcription-splicing coupling

$p_{n,1D3D}$		$p_{n,1D3D} = N_0 \Big/ \left(N_0 + k_{off,n}\left(n/k_E + n^2/x_d + \tau_t/n\right)\right)$	Probability of snRNP at IEJn	**Eq12**	**F2D, Eq11**	
N_0	Molecules		Number of freely diffusing snRNP	**Eq11**	**Eq11-12**	
$p_{n,3D}$		$p_{n,3D} = N_0 \Big/ \left(N_0 + k_{off,n}\left(n/k_E + \tau_t\right)\right)$	Probability of snRNP at IEJn considering only 3D diffusion	**Eq12**	**F2D**<br>**Eq12**	
$S_{s,n}$		$S_{s,n} = 100\int_0^n p_{m,1D3D}\,dm \Big/ n$	Splicing efficiency	**Eq13**	**F5**<br>**Eq13**	
μ	Bases		Value that maximizes $S_{s,n}$	**After Eq13**	**F6**	
${}_{c,k}v_i$			Signal from exon at base *i*, transcript *c*, tissue *k*	**After Eq13**		Note: this is different from $s_{e,c,k}$
$g_{c,k}$	Molar	$g_{c,k} = \int_0^n {}_{c,k}v_i\,di \Big/ n$	Transcript signal	**After Eq13**		
c	index		Transcript index	**Eq 14**	**Eq14**	
k	index		Tissue index	**Eq 14**	**Eq14**	
ε	index		Exon index	**Eq 14**	**Eq14**	
$s_{\varepsilon c,k}$	Relative units		Log2 signal exon ε, transcript *c*, tissue *k*	**Before Eq 14**	**Eq14**	
m_c			# exons in transcript *c*	**Eq 14**	**Eq14**	
$\pi_{\varepsilon,c,k}$	probability	$\pi_{\varepsilon,c,k} = \dfrac{s_{\epsilon,c,k}}{\sum_{i=1}^{m_c} s_{i,c,k}}$	Probability that exon ε is included in the final transcript	**Before Eq 14**		
$\sigma_{\varepsilon k,c}$	Index	$\sigma_{\varepsilon,c,k} = s_{\varepsilon,c,k} \big/ g_{c,k}$	Splicing index for exon ε in transcript *c* and tissue *k*.	**Before Eq 14**	**Eq14**	
Γ_c		$\Gamma_c = \sum_{\varepsilon=1}^{m_c}\left(\sum_k\left(\sigma_{\varepsilon,k,c}\right)^2 - \left(\sum_k \sigma_{\varepsilon,k,c}\right)^2\right) \Big/ k$	Variance based scoring metric	**Eq14**	**Eq14**	
$f_{\varepsilon,k}$		$f_{\varepsilon,k} = \sum_c \left(100\left(s_{\varepsilon,c,k} - s_{1,c,k}\right) \big/ s_{1,c,k}\right)$	FENAS (first-exon normalized signal)	**Eq15**	**F4**<br>**Eq15**	
		$n = \theta(\varepsilon)$	Position of the εth exon	**Before Eq16**	**F3C-D**	
$h_{m,k}$	Relative units	$h_{m,k} = \sum_{r=1}^{b}\left(\sum_{\varepsilon=1}^{m} s_{\varepsilon,r,m,k} \big/ m\right) \Big/ b$	Genome level avrage of transcripts with m exons in kthth tissue	**Eq16**	**F6**<br>**Eq16**	
$b(m)$			Number of transcripts with *m* exons	**Eq16**	**Eq16**	
